# NR2F6 as a Prognostic Biomarker in HNSCC

**DOI:** 10.3390/ijms21155527

**Published:** 2020-08-01

**Authors:** Luise Klapper, Julika Ribbat-Idel, Patrick Kuppler, Finn-Ole Paulsen, Karl-Ludwig Bruchhage, Dirk Rades, Anne Offermann, Jutta Kirfel, Barbara Wollenberg, Christian Idel, Sven Perner

**Affiliations:** 1 Institute of Pathology, University of Luebeck and University Hospital Schleswig-Holstein, 23538 Luebeck, Germany; luise.klapper@student.uni-luebeck.de (L.K.); Julika.Ribbat-Idel@uksh.de (J.R.-I.); patrick.kuppler@student.uni-luebeck.de (P.K.); finnole.paulsen@student.uni-luebeck.de (F.-O.P.); Anne.Offermann@uksh.de (A.O.); Jutta.Kirfel@uksh.de (J.K.); or sperner@fz-borstel.de (S.P.); 2 Department of Otorhinolaryngology, University of Luebeck and University Hospital Schleswig-Holstein, Campus Luebeck, 23538 Luebeck, Germany; Karl-Ludwig.Bruchhage@uksh.de; 3 Department of Radiation Oncology, University of Luebeck and University Hospital Schleswig-Holstein, 23538 Lübeck, Germany; Dirk.Rades@uksh.de; 4 Department of Otorhinolaryngology, MRI Technical University Munich, 81675 Munich, Germany; barbara.wollenberg@tum.de; 5 Pathology, Research Center Borstel, Leibniz Lung Center, 23845 Borstel, Germany

**Keywords:** HNSCC, NR2F6, local recurrence, prognosis, recurrence-free survival, biomarker, immune checkpoint, TMA, FFPE

## Abstract

Head and neck squamous cell carcinoma (HNSCC)is the 6th most common cancer in humans worldwide and is associated with a poor prognosis for patients. NR2F6 has been identified as an immune checkpoint molecule in tumor-infiltrating T lymphocytes and is associated with a poor prognostic outcome in various cancers. The prognostic value of NR2F6 in HNSCC has not been described yet. We used a large, representative and clinically well-characterized cohort of 383 HNSCC patients, of which 22.4% developed a local recurrence. The NR2F6 expression was analyzed by using immunohistochemistry and was afterward correlated with clinical characteristics and clinicopathological features of HNSCC patients. Primary tumors from patients who develop a local recurrence have a higher NR2F6 expression than primary tumors which do not develop a local recurrence. Furthermore, a high NR2F6 expression is associated with poorer recurrence-free survival, although there is no correlation with overall survival. NR2F6 expression is independent of the T stage and UICC stage. NR2F6 might be a new prognostic biomarker for the early detection of local recurrences in HNSCC patients. Therefore, it may help to improve the recognition of patients who would benefit from more frequent follow-up examinations.

## 1. Introduction

Head and neck squamous cell carcinoma (HNSCC) is the sixth most common cancer in humans worldwide [1,2]. Surgery and/or chemoradiotherapy are the most common therapeutic options but are often linked to side effects, which are hard to endure for patients. They suffer from functional impairment such as dysphagia or permanent voice changes, as well as scars and visible deformations or chronic pain after surgery. The chemoradiotherapy may lead to xerostomia, necrosis, and fibrosis of the bone and soft tissue in the head and neck region [3]. Despite all therapeutic approaches, the prognosis for HNSCC patients is still rather poor, and the side-effects are severe. The majority of patients are initially diagnosed in advanced stages, i.e., UICC III and IV. With higher stages, the survival rates decrease significantly. The two-year survival rate for patients diagnosed with UICC stage III and IV tumors is only at nearly 30%, while 30–50% develop a local recurrence [2,4,5,6]. New therapeutic approaches, like immune checkpoint therapy or changes in the therapy regimen, have not improved this fate significantly. Additionally, the question of whether one should use neoadjuvant or adjuvant chemotherapy is still controversial [4,7,8,9].

Early identification of primary tumors that will develop a local recurrence is difficult and does not always depend on the UICC stage or tumor size. We aim to find new independent prognostic biomarkers, which may help to risk-stratify patients at the time of diagnosis and therefore to identify patients whose tumors will develop a local recurrence. These patients could be included in more frequent follow-up schedules in order to diagnose and therefore treat local recurrences earlier than in the regular follow-up schedules.

NR2F6 (nuclear receptor subfamily 2, group F, member 6), also known as Ear-2 or COUP-TFIII, is an orphan nuclear receptor. It regulates many biological and embryological processes, such as organogenesis and cellular differentiation [10,11]. It is also known for its functions as a transcription factor, coactivator, and repressor for the expression of various genes, including hormones, hormone receptors, and apolipoproteins [12,13,14,15,16,17]. In immune cells, it helps to regulate the expression of cytokines like Interleukin-21 (IL-21) or Interferon-γ (IFN-γ) [18,19], and it is also a negative regulator of T-cell development [20]. NR2F6 was defined as an intracellular immune checkpoint in tumor-infiltrating T-cells. It correlates with the expression of programmed cell death-1 (PD-1), programmed cell death ligand-1 (PD-L1), and cytotoxic T lymphocyte-associated protein 4 (CTLA-4), and it was assumed to be a potential target for immunotherapy [21,22,23]. However, NR2F6 has not only been described in tumor-infiltrating lymphocytes, but also in tumor cells of different solid cancer types, e.g., hepatocellular carcinoma, ovarian cancer, cervical cancer, and colon cancer. For example, it is associated with the occurrence of pelvic lymph node metastases in cervical cancer and with poorer overall survival (OS) and cisplatin resistance in ovarian cancer [24,25,26,27].

Herein, we investigated, in particular, the characteristics of NR2F6 expression in HNSCC tissue, as well as the relationship between NR2F6 expression and the clinicopathological data of HNSCC patients, and we explored the value of NR2F6 as a prognostic factor.

## 2. Results

**Figure 1:**
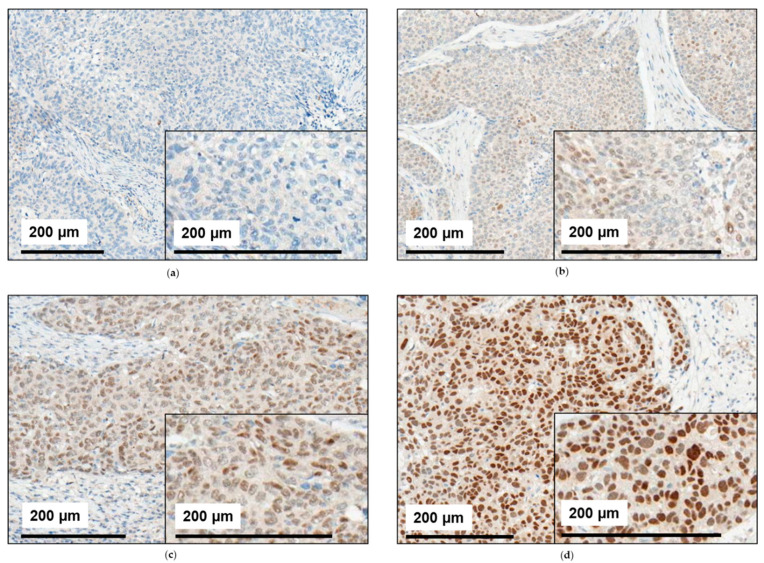
Staining patterns for NR2F6 in HNSCC. The HNSCC tissue from our cohort was immunohistochemically assessed for NR2F6 expression. (**a**) Some tumors showed no NR2F6 expression. In other tumors, the mean NR2F6 expression was (**b**) low, (**c**) medium, or (**d**) high. Some HNSCC also showed an inhomogeneous NR2F6 expression with (**b**,**c**) positive and negative cells.

Immunohistochemically stained HNSCC tissue showed a broad variety of staining patterns. While some tissue samples were stained completely negative for NR2F6 (Figure 1a), others were stained partly (Figure 1b,c) or completely positive (Figure 1d). The overall staining intensity varied between different tissue samples, reflecting an intercase heterogeneity, but also in between the nuclei of distinct cancer tissues, reflecting an intracase heterogeneity (Figure 1a–d).

### 2.1. NR2F6 Expression in Primary Tumors, Lymph Node Metastases, Distant Metastases, and Local Recurrences

**Figure 2:**
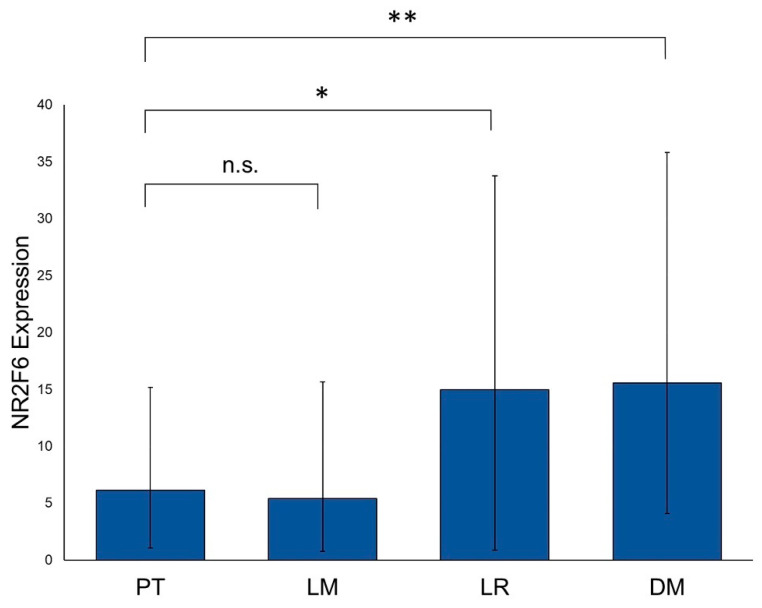
NR2F6 expression in primary tumors, metastases, and local recurrences. HNSCC tissue samples of PTs (*n* = 330), LMs (*n* = 127), LRs (*n* = 62), and DMs (*n* = 21) were assessed for their NR2F6 expression levels. NR2F6 expression in PTs is significantly lower than in LRs (Mann–Whitney test, *p* = 0.014) and DMs (Mann–Whitney test, *p* = 0.005). (* *p* ≤ 0.05; ** *p* ≤ 0.01; n.s. = not significant).

We analyzed the NR2F6 expression in the tissue of primary tumors (PTs; *n* = 330), lymph node metastases (LMs; *n* = 127), distant metastases (DMs; *n* = 21), and local recurrences (LRs; *n* = 62). PTs showed the lowest NR2F6 expression levels. LMs had also low levels of NR2F6 and were not significantly differentiated from PTs. NR2F6 was expressed significantly higher in DMs (*p* = 0.005) and LRs (*p* = 0.014) when compared to PT tissue (Figure 2).

### 2.2. NR2F6 Expression in Different HNSCC Tumor Sites

**Figure 3:**
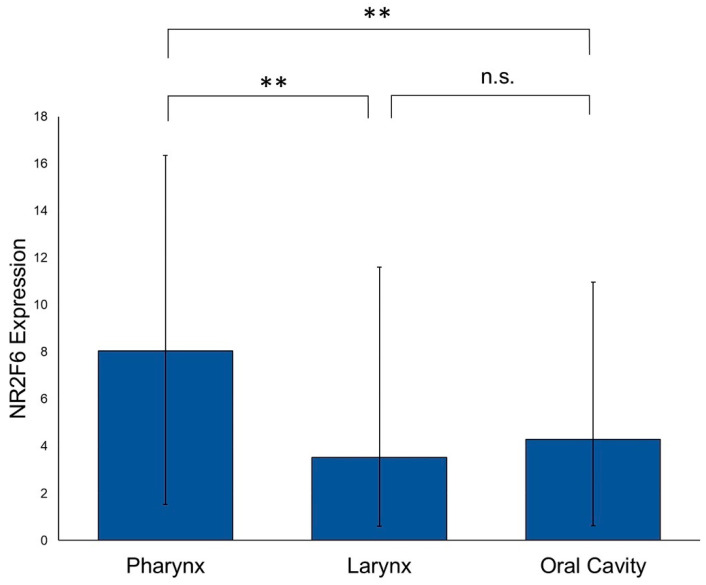
NR2F6 expression in different HNSCC tumor sites. The NR2F6 expression is significantly higher in pharyngeal HNSCCs than in laryngeal (Mann–Whitney test, *p* = 0.008) and oral cavity HNSCCs (unpaired *t*-test, *p* = 0.004). There is no significant difference between laryngeal and oral cavity HNSCCs. (** *p* ≤ 0.01; n.s. = not significant).

In the next step, we examined if there are differences in the NR2F6 expression levels between different sites of origin in primary HNSCCs. We used PT tissue that originated from the oropharynx (*n* = 103), hypopharynx (*n* = 45), larynx (*n* = 96), and oral cavity (*n* = 76). The NR2F6 expression in oropharyngeal and hypopharyngeal HNSCCs was nearly equal, with no statistically significant differences. Consequently, oropharyngeal and hypopharyngeal HNSCCs were pooled as pharyngeal HNSCCs. The NR2F6 expression was higher in pharyngeal HNSCCs (*n* = 148) than in laryngeal HNSCCs (*p* = 0.008) and oral cavity HNSCCs (*p* = 0.004). Laryngeal HNSCCs showed the lowest median NR2F6 expression level. There was no statistically significant difference in the NR2F6 expression between laryngeal and oral cavity HNSCCs (Figure 3).

### 2.3. NR2F6 Expression Is Higher in Primary HNSCCs with Immune Cell Infiltration than in Primary HNSCCs without Immune Cell Infiltration

**Figure 4:**
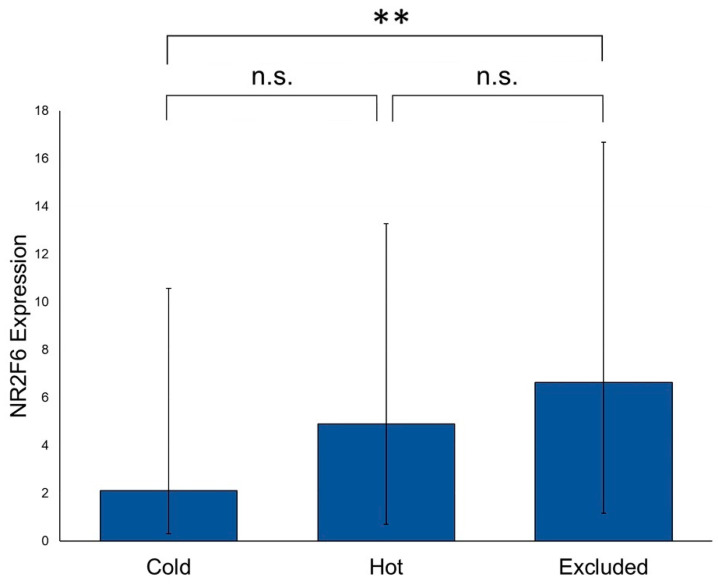
NR2F6 expression in PTs with and without immune cell infiltration. There was a significant difference in the NR2F6 expression between “cold” and “excluded” PTs (Mann–Whitney test, *p* = 0.006). “Cold” and “hot” PTs and “hot” and “excluded” PTs showed no significant differences, but a trend was visible. (** *p* ≤ 0.01; n.s. = not significant).

PTs of our cohort were assessed for the presence or absence of immune cell infiltration. The immune cell infiltration in “hot” tumors is located diffusely within the whole tumor, whereas the immune cells in “excluded” tumors can just be found in the peritumoral stroma. “Cold” tumors do not show any kind of immune cell infiltration. (For further details, see Ribbat–Idel et al. [28]). The highest NR2F6 expression was detected in “excluded” PTs, followed by “hot” PTs. “Cold” PTs showed the lowest NR2F6 expression. We first analyzed the difference in the NR2F6 expression in “cold” tumors vs. “hot” and ”excluded” tumors (tumors without any immune cell infiltration vs. tumors with immune cell infiltration). We found a statistically significant difference between these two groups (*p* = 0.01). In further analyses, the difference between “cold” and “excluded” PTs was statistically significant (*p* = 0.006), whereas the differences between “hot” and “cold” versus “hot” and “excluded” PTs were not statistically significant. However, a trend was visible (Figure 4).

### 2.4. NR2F6 Expression Is Higher in Primary HNSCCs with Subsequent Local Recurrences

**Figure 5:**
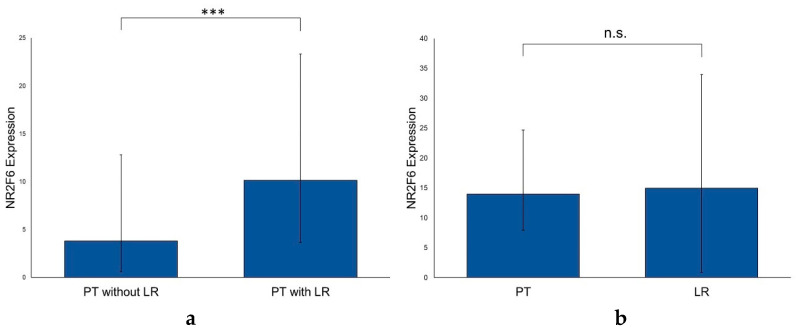
Correlation of NR2F6 expression in PTs with and without LRs and NR2F6 expression in PTs with matching LRs. (**a**) The NR2F6 expression in PTs with LRs was significantly higher than in PTs without LRs (Mann–Whitney test, *p* < 0.001). (**b**) The NR2F6 expression in PTs and matched LRs was not statistically significant (Wilcoxon signed-rank test, *p* = 0.664). (*** *p* ≤ 0.001; n.s. = not significant).

74 HNSCC patients of our cohort developed an LR, whereas 256 HNSCC patients did not. The NR2F6 expression in primary HNSCCs with subsequent LRs was significantly higher than in HNSCCs without an LR (*p* < 0.001) (Figure 5a). In a matched-pair analysis between PTs with their LRs (*n* = 41) there was no significant difference in the NR2F6 expression (*p* = 0.664) (Figure 5b). We further analyzed whether the NR2F6 expression still correlated with the occurrence of an LR, depending on the tumor sites (pharynx, larynx, and oral cavity). The NR2F6 expression was significantly higher in PTs with an LR, independently of the location of the PT (Table S1). We detected no significant differences in the NR2F6 expression between PTs with or without LMs (*n* = 135 and *n* = 144, respectively) and PTs with or without DMs (*n* = 36 and *n* = 243) (*p* > 0.05).

### 2.5. NR2F6-Expressing HNSCCs Are Associated with a Short Recurrence-Free Survival

**Figure 6:**
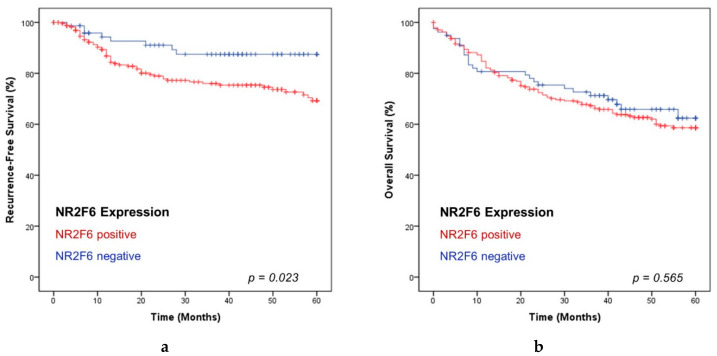
Kaplan Meier analyses of NR2F6 positive and negative HNSCC. (**a**) NR2F6 positive HNSCCs correlated with a significantly lower rate in RFS (*p* = 0.023). (**b**) There was no significant difference in the OS rates between NR2F6 positive and negative tumors (*p* = 0.565).

To detect differences in the OS and recurrence-free survival (RFS) between NR2F6 negative and positive tumors, the Kaplan Meier method and log-rank testing were applied. Patients with NR2F6 negative HNSCCs had a significantly longer recurrence-free survival than patients with NR2F6 positive HNSCCs (log-rank test, *p* = 0.023) (Figure 6). The cumulative rates of five-year recurrence-free survival were 87.5% for patients with NR2F6 negative HNSCCs, and 69.3% for those with NR2F6 positive HNSCCs. Univariate and multivariate cox regression analyses were performed to state whether the NR2F6 expression was a significant prognostic factor for recurrence-free survival and independent from other prognostic factors (Table S2). The univariate analyses revealed that the NR2F6 expression (Hazard Ratio (HR) = 2.296; *p* = 0.028), T stage (HR = 1.954; *p* = 0.002), UICC stage (HR = 1.685; *p* = 0.021), and p16 expression (HR = 0.430; *p* = 0.003) were significant prognostic factors. In the multivariate analysis, the NR2F6 expression (HR = 2.551; *p* = 0.014) and p16 expression (HR = 0.342; *p* = 0.007) were determined as independent prognostic factors for the recurrence-free survival of HNSCC patients. We found no significant difference in the OS rates between patients with positive and negative NR2F6-expressing PTs (*p* > 0.05) (Figure 6). There were also no significant differences in OS and RFS between NR2F6 negative, low, medium, and high expressing PTs (Figure S1).

### 2.6. The NR2F6 Expression Is Independent of T and UICC Stages and the p16 Status of PTs

**Figure 7:**
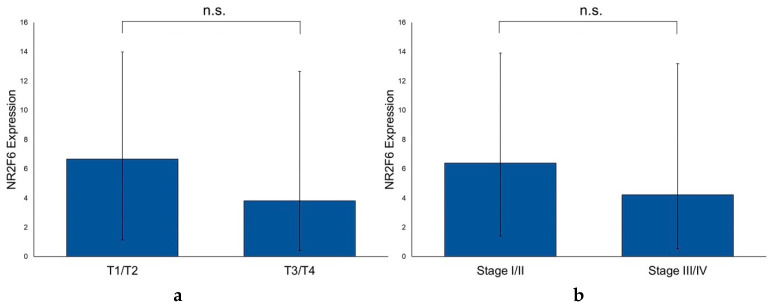
Differences in the NR2F6 expression of different T stages and UICC stages of HNSCC PTs. (**a**) The NR2F6 expression in PTs did not correlate with the T stage (Mann–Whitney test, *p* = 0.718). (**b**) The NR2F6 expression in PTs did not correlate with the UICC stage (Mann–Whitney test, *p* = 0.474). (n.s. = not significant).

We compared the NR2F6 expression in PTs of different T and UICC stages. We could show that the NR2F6 expression was independent of the T stage and UICC stage, as no significant differences were found between stages T1 and T2 versus stages T3 and T4 (*p* = 0.718) or between UICC stages I and II versus III and IV (*p* = 0.474), respectively (Figure 7). Furthermore, we analyzed the correlation between NR2F6 expression in PTs and the age, sex, alcohol abuse, nicotine abuse, and p16 status of the patients, but no significant correlations were found either (Table S3).

## 3. Discussion

HNSCC is the sixth most common cancer in humans and still has a rather poor prognosis. The introduction of blocking antibodies against PD-1 in the treatment of recurrent HNSCCs has improved overall survival, but the improvement has been inferior when compared to other malignancies like melanoma [29]. The standard treatment of primary HNSCCs is a primary combination of chemoradiotherapy or surgery. The latter may then be complemented by risk-adapted irradiation with or without chemotherapy [29]. Within the group of HNSCCs, tumors may originate from different locations, i.e., the oral cavity, oropharynx, hypopharynx, and larynx. We start to understand that HNSCCs differ in tumor biology depending on the site of origin. The clearest difference is seen in oropharyngeal cancers, in which the human papillomavirus (HPV) has a huge impact on the overall survival of patients. In the oral cavity, the hypopharynx, and the larynx, no impact on the overall survival by HPV has been detected so far [30]. Moreover, the response towards irradiation is also dependent on the site of origin. PTs of the hypopharynx have the worst response towards radiotherapy [31].

The differences in HNSCCs, defined by the site of origin, can also be shown by the data presented here. HNSCCs of the oral cavity and the larynx have a significantly lower NR2F6 expression than pharyngeal HNSCCs; meanwhile, in this group, there was no difference between oro- and hypopharyngeal HNSCCs. The higher expression in the pharyngeal HNSCCs cannot be explained by HPV infection because the NR2F6 expression was independent of the p16 expression. Additionally, as long as we do not know the exact functions of NR2F6 in cancer cells, the different expression patterns are an indicator of a different tumor biology. Despite the differences in the overall NR2F6 expression at the different tumor sites, NR2F6 seems to be upregulated in the PTs with an LR, independently of the mean expression level at the tumor site. Even in the NR2F6 lower-expressing laryngeal and oral cavity HNSCCs, an increase of NR2F6 in PTs with an LR could be seen. For clinical diagnostics, it might be necessary to define particular thresholds for the assessment of the NR2F6 expression for every HNSCC tumor site.

Not only is the site of origin related to the NR2F6 expression, but so is the immune status of a tumor. With the introduction of immune checkpoint inhibitors, researchers focused more on the immune status of tumors [28,32,33,34]. The simple categorization of tumors into those with immune cell infiltration and those without immune cell infiltration shows that there is also a significant difference in the NR2F6 expression. In lung cancer, it was shown that NR2F6 was expressed in lymphocytes and not in cancer cells. The higher NR2F6 expression in these lymphocytes correlated with a higher PD-1 and CTLA-4 expression in the lymphocytes, as well as PD-L1 expression in tumor cells. It has been identified as an immune checkpoint molecule and as a potential new therapeutic target for immunotherapy [21]. Interestingly, in all HNSCC types analyzed here, there was no detectable expression in lymphocytes. NR2F6 was only expressed in the cancer cells, but there was still a significantly higher expression in the PTs with immune cell infiltration. Further investigation is needed to elucidate if this correlation has a functional connection or if it is a surrogate association, comparable to the expression of p16 in HPV-associated HNSCCs.

Furthermore, without fully understanding the complete function of NR2F6 in HNSCC cells, the expression of NR2F6 within cancer cells instead of immune cells has been shown before in other solid cancer types (e.g., colon cancers) [24,25,26,27]. In most of these cancers, a higher NR2F6 expression was associated with a poor prognosis. We could not see an effect of the NR2F6 expression on the OS of patients. This result could be explained by the increased overall morbidity of HNSCC patients. More than 80% of patients in our cohort consumed nicotine, and over 40% consumed alcohol, both of which are known for their many harmful effects, leading to several comorbidities. These toxins may also impact the poor survival of HNSCC patients [35,36].

Nevertheless, we showed that, in HNSCCs, the NR2F6 expression was associated with a decreased RFS. Additionally, the PTs that developed an LR had a higher NR2F6 expression already. The expression level of NR2F6 did not correlate with the T stage, UICC stage, or p16 expression of PTs and was stated as an independent prognostic factor for the RFS of HNSCC patients. Therefore, NR2F6 is independent of the three established prognosis markers in HNSCCs [37]. The question of whether NR2F6-overexpressing primary HNSCCs might need a more intensified primary therapy cannot be answered at this point. We do not yet know if these tumors are more prone to radiation or chemotherapy. To answer this question, a more functional analysis and subsequent prospective studies will be needed. However, NR2F6 might close a gap for a better risk stratification of HNSCCs. In daily clinical work, we see patients with PTs of high tumor stages who never develop an LR. On the other hand, we see patients with a low tumor stage who develop a very early LR despite aggressive treatment. The NR2F6 expression in the PTs might serve as a stratification marker to predict the risk of an LR. Those patients with an NR2F6 expression in the PT might need follow-up investigations more frequently and with shorter periods between appointments. This may help to detect LRs earlier and to treat cancer at the earliest possible time. So far, the follow-up schedules after the primary treatment may vary in different hospitals and cancer centers. In the first year, the patients receive a clinical examination every six to 12 weeks. After one year, the first CT/MRI scans and re-biopsies of the previous tumor area are performed, if the clinical examinations have been without pathological findings so far. Only with a clinical suspicion of an LR are CT/MRI scans and re-biopsies performed at an earlier date. Most LRs occur within the first 18 months after the primary treatment [2,4,5,6]. However, in our cohort, we observed patients with an LR at a later time as well. Therefore, a tighter schedule of follow-up examinations is not only necessary for the first 18 months after treatment. The problem regarding which kind of schedule is favorable in HNSCC patients with NR2F6 overexpression must be evaluated in a prospective study. A clinical examination in these patients every six weeks, combined with a CT/MRI scan and re-biopsy every six months in the first two years and a clinical examination every three months, with yearly CT/MRI scans and re-biopsies in the next three years might be conceivable. As this is associated with higher costs and risks for the patients (e.g., general anesthesia, contrast agents), a prospective study is needed to define a new follow-up schedule in NR2F6-overexpressing HNSCC.

## 4. Materials and Methods

### 4.1. Patient Data and Tumor Material

Our study protocol was approved by the Ethics Committee of the University of Luebeck (project code AZ 16-277, date of approval 18 November 2016) and conducted in accordance with the Declaration of Helsinki. We used our vast and well-characterized HNSCC cohort for the analysis of NR2F6 expression. Our group established a retrospective and anonymized cohort of 383 Patients with HNSCCs [28,38]. All patients were diagnosed between 2012 and 2015 in our Institute of Pathology and treated in the on-campus Department for Otorhinolaryngology of the University Medical Center Schleswig-Holstein in Luebeck. All clinical data were obtained from medical records. 22.1% were female, and 77.6% were male. 81.1% of all patients consumed nicotine (at least one pack per year), and 40.8% consumed alcohol, whereas 11.5% and 53.9% did not consume nicotine or alcohol, respectively. 44.8% of all PTs were pharyngeal HNSCCs (including 13.6% hypopharyngeal HNSCCs and 31.2% oropharyngeal HNSCCs), 29.1% were laryngeal HNSCCs, 23.0% were HNSCCs of the oral cavity, and 3% were located in other regions of the head and neck or were cancers of unknown primary (CUP). 73.9% of all PTs were p16 negative, and 26.1% were p16 positive. 77.6% of all patients did not develop and 22.4% did develop an LR. At the time of the first-time diagnosis, 50.0% of PTs were classified as T stage 1/2 and 49.4% as T stage 3/4; 37.6% were classified as UICC stage I/II and 62.1% as UICC-stage III/IV. TNM stages were assessed by the 8th edition of the TNM classification for HNSCC. We used tissue of PTs (*n* = 330), LMs (*n* = 127), DMs (*n* = 21), and LRs (*n* = 62) for analyzation of the NR2F6 expression.

Formalin-fixed paraffin-embedded (FFPE) tumor tissues were sampled and arranged to form tissue microarrays (TMAs), as previously described [39]. The tissue of PTs, LMs, DMs, and LRs were used. Each TMA consisted of triplet cores of 1 mm^2^ from up to 54 tumor samples and up to six samples of benign tissue from the head and neck region. For a statistical evaluation, we analyzed three core samples from each tumor and calculated the mean value.

### 4.2. Immunohistochemistry and Evaluation of Stained Slides

We performed immunohistochemical staining on 4-µm thin sections of FFPE tissue after it was deparaffinized and antigens were heat-mediated retrieved, as previously described [40]. For the anti-NR2F6 antibody (rabbit polyclonal, 1:50, ab137496, Abcam, Cambridge, UK), the IView DAB Detection Kit was used on a Ventana BenchMark automated staining system (Roche, Basel, Switzerland), as previously described [41].

NR2F6 stained slides were scanned and digitalized by using the Ventana iScan HT scanner (VentanaTuscon, AZ, USA). For the digital quantified analysis, the semi-automatic image analysis software Definiens Tissue Studio (Definiens Developer XD 2.0, Definiens AG, Munich, Germany) was used. With this tool, the analysis of the staining intensities in different parts of the cell is possible. To exclude benign tissue and stromal cells, tumor cell areas were manually annotated as a region of interest (ROI) for each TMA core by a board-certified pathologist. The brown staining intensity of tumoral nuclei in the annotated area reached a readout with a range from 0.012–0.734. Staining thresholds were set to differ between negative and positive nuclei. The proportion of positive tumor cells in all tumor cells was detected automatically as a positive index (readout from 0–100). This positive index was used to reflect a staining homogeneity in further analyses.

The immunohistochemical staining of our HNSCC cohort showed a high variation of the staining intensity and the number of stained tumor cells within the tumor tissue. Some HNSCCs were stained exclusively positive, while some others were stained exclusively negative for NR2F6. Other tumors included both negative cells and positive cells with varying staining intensities. The positive index and the staining intensity were multiplied and transformed into an expression index (score from 0–71.25), which displayed the NR2F6 staining intensity, as well as the heterogeneity of the tumor. For further analyses, we exclusively employed the NR2F6 expression index to describe the NR2F6 expression in HNSCC tissues. An expression index between 0 and 1 was considered negative, whereas an expression index >1 was considered positive. The positive expression indices were divided into the subgroups low (expression index 1–6), medium (expression index 6–17), and high (expression index 17–72). All steps of the digital analysis, except for slide scanning, were performed on the same computer (Windows 7 based, 24” monitor, resolution 1920 × 1080 px.). This approach was previously published and used by our group [38,42].

### 4.3. Pathological Evaluation of Immune Cell Infiltration

PTs of our cohort were assessed for the presence or absence of immune cell infiltration. HNSCC tissues were stained in hematoxylin and eosin (HE). The tissue was then examined by a board-certified pathologist and classified into the established categories “cold”, “hot”, and “excluded”. “Cold” tumors are defined as tumors without any immune cell infiltration, whether it be intratumoral or peritumoral stroma tissue. The subgroup of “hot” tumors is characterized by immune cell infiltration within the tumor. In “excluded” tumors, the immune cells are exclusively infiltrating the peritumoral stroma at the tumor boundaries.

### 4.4. Statistical Analysis and Visualization

We used IBM SPSS Statistics for Windows (SPSS Statistics, v. 24, IBM Corp., Armonk, NY, USA) for the statistical analyses. We applied Mann–Whitney tests to compare the NR2F6 expression in different tissue types and at different locations of the PT, different UICC stages, clinicopathological features, and to compare between PTs with and without LMs, LRs, and DMs. A Wilcoxon signed-rank test was applied to compare NR2F6 expression in PTs and matched LRs. The Kaplan–Meier method and log-rank testing were used to calculate 60-months OS, PFS, and RFS, and to test for statistical significance. *p*-values lower than 0.05 were considered statistically significant. The bar charts show the median NR2F6 expression, and the error bars reflect the 25th and 75th percentile of the NR2F6 expression.

We used the following software to create artwork and edit photomicrographs: Inkscape (v. 0.92.5, The Inkscape Project c/o Software Freedom Conservancy, Brooklyn, NY, USA, https://inkscape.org/), GIMP (v. 2.10.18, The GIMP Project c/o GNOME Foundation, Orinda, CA, USA, https://www.gimp.org), and Microsoft Powerpoint (Microsoft Office 365 ProPlus, Microsoft, Redmont, Washington, DC, USA).

## Supplementary Materials

Supplementary materials can be found at https://www.mdpi.com/1422-0067/21/15/5527/s1.

## Abbreviations

CTLA-4	Cytotoxic T lymphocyte-associated protein 4
DM	Distant metastasis
FFPE	Formalin-fixed paraffin-embedded
HE	Hematoxylin-eosin
HNSCC	Head and neck squamous cell carcinoma
HPV	Human papillomavirus
HR	Hazard Ratio
IFN-γ	Interferon-γ
IL-21	Interleukin-21
LM	Lymph node metastasis
LR	Local recurrence
NR2F6	Nuclear receptor family 2 subgroup 6
PD-1	Programmed cell death-1
PD-L1	Programmed cell death ligand-1
PT	Primary tumor
ROI	Region of Interest
TMA	Tissue microarray
