# Supplementary material for: NR2F6 as a Prognostic Biomarker in HNSCC
Source: Int J Mol Sci. 2020 Aug 1;21(15):5527. doi: 10.3390/ijms21155527 (PMC7432340; doi:10.3390/ijms21155527)
Supplement: Supplementary file 1 [file ijms-21-05527-s001.pdf]

International Journal of
Molecular Sciences

MDPI

Supplementary Materials

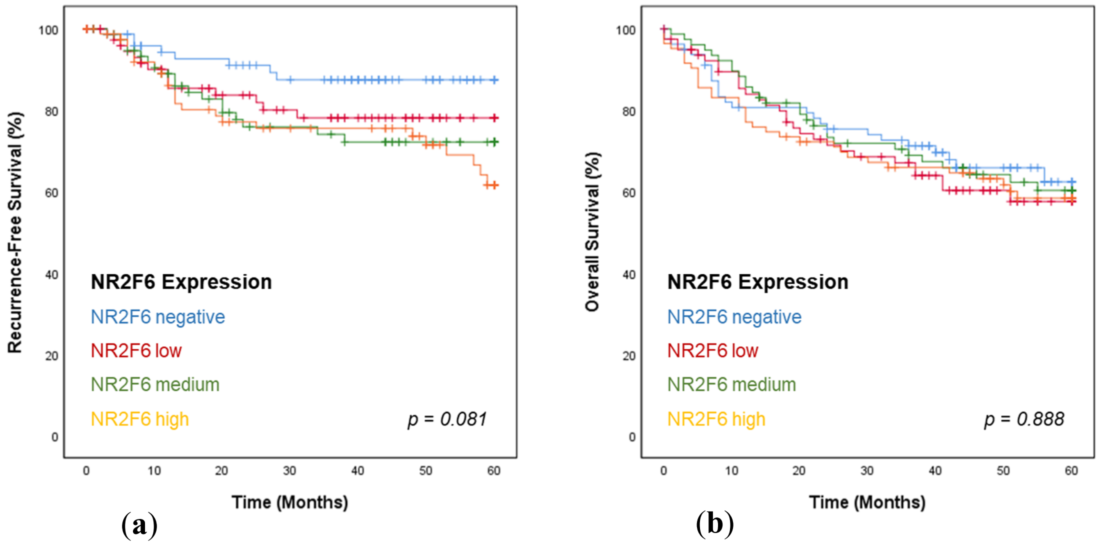

(**a**) (**b**)

Figure S1. Kaplan Meier Analysis for negative, low, medium, and high NR2F6-expressing HNSCC. There are no significant differences in the (**a**) RFS ($p = 0.081$) and (**b**) OS ($p = 0.888$) of HNSCC patients with NR2F6 negative, low, medium, and high expressing PTs.

Table S1. Correlation of LRs and the NR2F6 expression in PTs at different tumor sites (Mann–Whitney test, * $p \leq 0.05$; ** $p \leq 0.01$).

Tumor Site	LR	Number of Patients	Median NR2F6 Expression	Mean NR2F6 Expression	*p*-value
**Pharynx**	No	120	8.146	10.906	0.014 *
	Yes	28	16.457	16.483	
**Larynx**	No	79	3.399	6.310	0.010 **
	Yes	34	9.949	13.718	
**Oral Cavity**	No	56	2.011	6.739	0.040 *
	Yes	20	8.622	9.813	

Table S2. Univariate and multivariate cox regression for 5-year RFS (* $p \leq 0.05$; ** $p \leq 0.01$)

Variable	Univariate Cox Regression			Multivariate Cox Regression		
	p-value	Hazard Ratio	95% Confidence Interval	*p*-value	Hazard Ratio	95% Confidence Interval
**NR2F6 expression**	0.028 *	2.296	1.092–4.824	0.014 *	2.551	1.211–5.374
**T Stage**	0.002 **	1.954	1.267–3.014	0.063	2.004	0.962–4.174
**UICC Stage**	0.021 *	1.685	1.082–2.625	0.819	0.910	0.404–2.047
**p16 expression**	0.003 **	0.430	0.246–0.751	0.007 **	0.342	0.156–0.748

Table S3. Correlation of the NR2F6 expression with clinicopathological features of HNSCC patients (Mann–Whitney test).

Clinicopathological Feature		Number of Patients	Mean NR2F6 Expression	Median NR2F6 Expression	*p*-value
**Age**	<61	152	9.95	6.91	0.496
	>61	176	9.52	4.45	
**Sex**	Female	73	9.83	6.20	0.965
	Male	256	9.70	5.62	
**Alcohol abuse**	Yes	142	9.41	7.09	0.465
	No	176	11.29	4.32	
**Nicotine abuse**	Yes	281	9.56	5.89	0.708
	No	32	9.02	4.46	
**p16 expression**	Yes	86	9.27	7.68	0.140
	No	244	11.13	5.07	
